# Gold complex QB1561 suppresses drug-resistant cancer cells by inhibiting TrxR and mitochondrial respiratory function

**DOI:** 10.3389/fphar.2025.1560880

**Published:** 2025-02-24

**Authors:** Hai-Ling Gao, Wenwen Ding, Zhi-Xin Shen, Qingbin Cui

**Affiliations:** ^1^ Department of Histology and Embryology, Shandong Second Medical University, Weifang, Shandong, China; ^2^ Department of Experimental Research, Sun Yat-sen University Cancer Center, Guangzhou, China; ^3^ Department of Thyroid and Breast Surgery, Affiliated Hospital of Shandong Second Medical University, Weifang, Shandong, China; ^4^ Department of Cell and Cancer Biology, University of Toledo College of Medicine and Life Sciences, Toledo, OH, United States

**Keywords:** multi-drug resistance, Gold(I) complex, anti-cancer, ROS, mitochondria, oxygen consumption rate

## Abstract

**Introduction:**

Multi-drug resistance (MDR) is one of the leading reasons that cause the failures of cancer treatment. Novel agents that may reverse MDR and neutralize drug-resistant cancer cells are highly desirable for clinical practice. The targeting of cellular redox homeostasis and/or mitochondria-mediated energy metabolism are promising strategies for the suppression of drug-resistant cancer cells. Based on the structure of mono-gold(I) complex auranofin (AF), a drug candidate under clinical trials for cancer, we synthesized a new dual-gold(I) complex QB1561 and tested if it can inhibit drug-resistant cancer cells overexpressing ATP-binding cassette (ABC) transporters. We also investigated if QB1561 could inhibit thioredoxin reductase (TrxR), a well-known target of AF and other gold complexes, and assessed its impact on mitochondrial respiration.

**Methodology:**

Cell viability of drug-resistant cells upon QB1561 alone or combined with topotecan and mitoxantrone was determined by MTS assay. The expression of ABC sub-family G member 2 (ABCG2) in the lung cancer cell line NCI-H460/MX20 after treatment with QB1561 was assessed by Western blot. The Vi-sensitive ABCG2 ATPase activity in the membrane vesicles of High Five insect cells, TrxR activity, and ROS production were measured following QB1561 treatment. Colony formation was used to assess QB1561’s anticancer potential. SeaHorce Seahorse XF Analyzers were used to measure the oxygen consumption rate (OCR).

**Results:**

QB1561 suppressed the proliferation of drug-resistant cancer cells overexpressing ABC transporters, with IC_50_ values ranging from 0.57 to 1.80 μM, which was more effective than AF. QB1561 was able to partially reverse the resistance of mitoxantrone and topotecan in lung cancer NCI-H460/MX20 cells which overexpressed ABCG2, without altering the expression levels of ABCG2. QB1561 suppressed the colony formation of NCI-H460/MX20 cells, probably via ROS induction due to TrxR inhibition. QB1561 also efficiently suppressed OCR, suggesting its inhibition on mitochondrial respiration.

**Conclusion:**

QB1561 was effective for the treatment of MDR in drug-resistant cancer cells. Its further evaluation could be useful for the design and development of more gold-based anticancer drugs.

## Introduction

Cancer treatment has been improved dramatically as a result of decades of active progression of advanced surgery, radiotherapy, and innovatory chemotherapy, including targeted therapy and immunotherapy. However, the emergence of multi-drug resistance (MDR) significantly undermines the effective treatment of recurrent and drug-resistant cancers (Siegel et al., 2023). MDR can be triggered by various mechanisms, including target mutation, reduced drug uptake or increased drug efflux, drug metabolism into inactive form or sequestration from its target, adaptation of energy metabolism, resistance to cell death, etc. (Assaraf et al., 2019; Cui et al., 2022b; Narayanan et al., 2020). It is well-recognized that the overexpression of ATP-binding cassette (ABC) transporters is one of the most common reasons for MDR that causes treatment failures of certain conventional and target chemotherapeutics (Nobili et al., 2020; Cui et al., 2019; Quan et al., 2019). Functioning primarilly as a defensive transporting pump, ABC sub-family G member 2 (ABCG2) can uptake its cellular substrates, including many cytotoxic agents such as anticancer drugs that are structure different, and then move them out of cancer cells, leading to acquired drug resistance (Chen, 2011; Kathawala et al., 2015; Falguieres, 2022; Fan et al., 2023). Also known as breast cancer-resistant protein (BCRP), clinical data have shown that the overexpression of ABCG2 contributes to resistance against various chemotherapeutic agents in patients with different types of cancer, including non-small cell lung cancer (Lemos et al., 2011), hepatocellular carcinoma (Chen et al., 2016), acute myeloid leukemia (Damiani et al., 2016), acute lymphoblastic leukemia (Suvannasankha et al., 2004), and other cancers types (Wang et al., 2017). ABCG2 expression induced resistance of both conventional anticancer drugs such as topotecan and doxorubicin (Guo et al., 2018), anthracycline-based chemotherapeutics (Westover and Li, 2015), etc., and certain targeted therapies such as tyrosine kinase inhibitors (TKIs) (Li et al., 2016b; Chen et al., 2015). Over the past two decades, several inhibitors have been widely studied and used in the laboratory, including fumitremorgin C (FTC), febuxostat, Ko143, and elacridar (Toyoda et al., 2019), while none of them has been tested in clinical trials as a specific ABCG2 inhibitor although they exert sensitizing effects to anticancer drugs that are ABCG2 substrates, most likely due to limited benefits to patients and, more importantly, unanticipated and intolerant toxic effects. Other ABCG2 regulators derived from natural products (Wu et al., 2011; Cui et al., 2018b), or from repurposed old drugs or especially some TKIs, are still in preliminary evaluations (Gao et al., 2023; Wang et al., 2020a; Zhang et al., 2019; Dong et al., 2022). Therefore, novel ABCG2 inhibitors or modulators with novel structures and/or mechanisms remain an unmet clinical need in treating ABCG2-overexpressing cancers.

**FIGURE 1:**
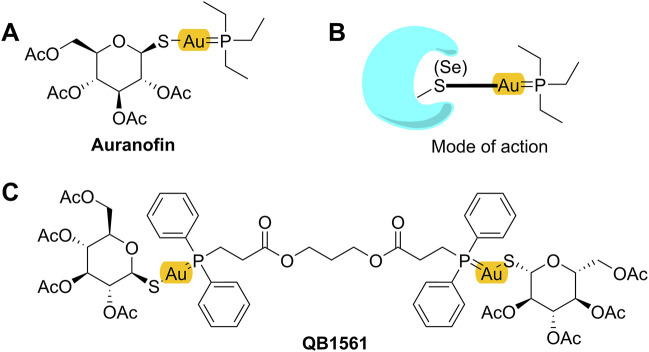
Mono-gold complex AF **(A)**, its projected mode of action **(B)**, and dual-gold(I) complex QB1561 **(C)**.

Recently, varied metal complexes, e.g., gold(I) complexes, have been found to be highly potent in suppressing the growth of cancer cells including drug-resistant cancer cells overexpressing ABC transporters or with other MDR mechanisms (Marmol et al., 2020; Abyar and Tabrizi, 2019; Chrysouli et al., 2018). Mono-gold complex, such as auranofin (AF) as shown in Figure 1A, is a drug candidate under clinical trials for cancer treatment. AF can bind to its target protein, the antioxidant thioredoxin reductase (TrxR), at the thiol (SH) or selenium (SeH) group (illustrated in Figure 1B), leading to the ceased TrxR function and, consequently, overproduced reactive oxygen species (ROS), which may finally lead to cell death (Pratesi et al., 2014; Marmol et al., 2019). AF is highly effective in multiple cancers, including non-small cell lung cancer cells (Li et al., 2016a). Additionally, there are also other types of gold(I) complexes that inhibited TrxR and showed strong anticancer effects, including an azolate gold(I) complex which showed ∼70 times higher cytotoxicity than cisplatin (Galassi et al., 2012), and other gold(I) complexes containing the key pharmacophore of AF but with different sulfur donors such as xanthate, thiocyanate, and cyanate, and they could suppress several cisplatin-resistant human cancer cells (Gandin et al., 2010).

While working as a candidate for certain cancers, AF itself is not highly potent in suppressing cancer cells, with IC_50_ values around several micromolars (Roder and Thomson, 2015; Cui et al., 2022a). Therefore, novel gold complexes with high potential are needed. Zorova et al. (2018) showed that, in addition to inhibt TrxR, AF also targeted the negative inner membrane of mitochondria (Griffiths, 2000; Zorova et al., 2018), likely through its active form ^+^Au(PEt_3_) as shown in Figure 1B (Pratesi et al., 2014; Zou et al., 2015). Thus, we hypothesized and validated that the installation of a dual-gold core (containing two Au ions) in the structure of gold complexes would facilitate the ability to target and accumulate in mitochondria (Cui et al., 2022b; Ding et al., 2023), thereby inhibiting their functions and initiating cell death. In this work, we investigated the anticancer activities of dual-gold complex QB1561 in drug-resistant cancer cells overexpressing ABC sub-family B member 1 (ABCB1) or ABCG2. QB1561 is a patented novel bi-gold(I) complex with a unique bi-phosphine ligand and an elongated linker through two ester bonds (Figure 1C). As anticipated, QB1561, with its higher gold atom content compared to AF, exhibits a 10-fold lower IC_50_ in A549 lung cancer cells (Supplementary Figure S1), likely due to its enhanced ability to target and accumulate in mitochondria. We reported here that QB1561 showed dual functions in drug-resistant cancer cells overexpressing certain ABC transporters. First, QB1561 was more potent than AF in suppressing the proliferation of drug-resistant cancer cell lines. Second, when combined with the chemotherapeutic agent and ABCG2 substrates mitoxantrone or topotecan, QB1561 could partially reverse resistance to these two drugs. This did not happen in combined treatment with paclitaxel. Mechanistic study indicated that these effects most likely happened by the dual inhibition of both TrxR and mitochondrial respiratory function. These data support the *in vivo* test of QB1561, and the information generated in this study may help direct the structural design of gold-based anticancer drugs.

## Materials and methods

### Synthesis of QB1561

The dual-gold(I) complex QB1561 was synthesized by a modified method following reported procedures (Cui et al., 2024; Ding et al., 2023). Chemical reagents were purchased and used without further purification. ^1^H-NMR, ^13^C-NMR and ^31^P-NMR spectra were recorded at ambient temperature on a 400 MHz spectrometer (AV-400 Bruker) in CDCl_3_. Chemical shifts are given in ppm (δ) referenced to CDCl_3_ with 7.26 for ^1^H and 77.16 for ^13^C. See the Supporting Materials for the NMR spectrum and details shifts.

### Cells and cell cultures

Human epidermoid carcinoma cell line KB-3-1 and its colchicine-selected ABCB1-overexpressing cell line KB-C2, cisplatin-resistant KCP4 cells, arsenic trioxide (ATO)-resistant KB-ATO cells, human lung cancer cell line NCI-H460, and its mitoxantrone-selected ABCG2-overexpressing NCI-H460/MX20 cells, human colorectal cancer cell lines, SW620 and adriamycin-selected ABCB1-overexpressing SW620/Ad300 cells were generously provided by Dr. Zhe-Sheng Chen (St. John’s University, NY, US). Cells were cultured in DMEM (Gibco-BRL, Paisley, United Kingdom) supplemented with 10% fetal bovine serum (FBS, Gibco-BRL, Paisley, United Kingdom). All cells were incubated in a humidified incubator at 37°C supplemented with 5% CO_2_.

### Cell viability

Cells were seeded in 96-well plates (3,000–4,000/well) and incubated overnight. Later the next day, QB1561 or AF and other associated drugs were added to each well at gradient concentrations and co-incubated for 72 h. MTS reagent (5 mg/mL, 20 μL) was added to each well and incubated for another 4 h at 37°C. For the reversal experiment, the cells were pre-treated with indicated concentrations of QB1561 for 2 h, and then mitoxantrone or topotecan were added to incubate for 72 h. The absorbance was measured under 490 nm by a microplate reader. Growth inhibition by a drug was presented by IC_50_, which was calculated using GraphPad Prism 8.4.0 software (San Diego, CA, United States). Experiments were carried out in triplicate.

### Western blot analysis

After treatment of QB1561 at indicated concentrations and time, cells were washed with ice-cold PBS and lysed with a protease inhibitor cocktail and a phosphatase inhibitor cocktail (Roche, Indianapolis, Indiana, United States) for 15 min on ice. Cell debris was removed by centrifugation at 12,000 g for 15 min at 4°C. The protein lysates were analyzed by standard SDS-PAGE and transferred to a nitrocellulose membrane. The primary antibody (1:800) and the secondary antibody labeled with Horseradish Peroxidase (HRP, 1:1,500, Cell Signaling Technology, Dancers, MA, United States) were used to determine the presence of protein, ABCG2 (BXP-21, Santa Cruz Biotechnology, TX, United States) and the internal control GAPDH (Thermo Fisher Scientific, Rockford, IL, United States).

### ATPase assay

The ATPase activity of vanadate-sensitive ABCG2 was measured as previously described (Ji et al., 2019; Gao et al., 2020). Briefly, 10 μg membrane (High five insect cells as the ABCG2 membrane vesicles) was incubated in assay buffer containing 50 mM MES (pH 6.8), 50 mM KCl, 5 mM sodium azide, 2 mM EGTA, 2 mM DTT, 1 mM ouabain, and 10 mM MgCl_2_. Then QB1561 was incubated with the membrane vesicles for 3 min. The ATP hydrolysis was initialized by adding 5 mM of Mg-ATP. After incubating at 37°C for 20 mins, the reaction was terminated by adding 100 μL 5% SDS solution. The inorganic phosphate (Pi) was measured at 880 nm using a spectrophotometer (Wang et al., 2020b).

### Colony formation assay

NCI-H460/MX20 cells in 2 mL DMEM medium with 10% FBS were seeded in a 6-well plate (500-600/well) and incubated overnight. Then, the cells were treated with QB1561 at 0.01, 0.03, 0.1, 0.3 and 1 μM for 14 days. Then the medium was removed, and the cells were washed gently with PBS twice (1 mL each time) and incubated with 1 mL MeOH for 15 min. Then, 1 mL crystal violet solution (Beyotime, Shanghai, China) was added to each well, incubated at room temperature for 30 min, and then washed with running water. After drying out, the colony number of each well was counted.

### Cellular TrxR activity

NCI-H460/MX20 cells were seeded in 6-well plate (5 × 10^5^/well) and incubated overnight before QB1561 and AF at 0.1 and 0.3 μM were added into each well and incubated for another 4 h. Thioredoxin Reductase Activity Colorimetric Assay Kit (Cat No. K763-100, Abcam) was used to examine the cellular TrxR activity as per manufacturer’s instruction (Cui et al., 2022a).

### ROS levels

After being treated with QB1561 for 4 h, NCI-H460/MX20 cells were collected and stained with H2DCFDA (Thermo Fisher Scientific, Rockford, IL, United States) at 1 μM for 2 or 6 h for the global ROS level. Then the cells were washed twice and re-suspended in PBS, followed by flow cytometry analysis using FACS Calibur flow cytometer (BD Biosciences, San Diego, CA, United States). The fluorescence intensity was normalized to the control group.

#### Cellular oxygen consumption (OCR) assay

The OCR was measured by the Seahorse Bioscience Extracellular Flux Analyzer (XF24, Seahorse Bioscience, Billerica, MA, United States) following the manufacturer’s instructions. The XF Assay Media was supplemented with glucose (5.5 mM) and pyruvate (1 mM), and the pH was adjusted to 7.4. Oxygen consumption was recorded every eight minutes after gradual additions of QB1561 or auranofin at 1, 3, and 5 μM. Oligomycin (5 μM), FCCP (1 μM), and antimycin A/rotenone (0.5 μM) were added. After the addition of QB1561 or AF, basal respiration was measured. Coupled respiration was expressed as the decrease from basal respiration after the addition of oligomycin. Maximal respiration was taken as the highest measurement after the addition of FCCP (Meng et al., 2019; Cui et al., 2022b).

## Statistics

The results in this study were presented and analyzed using Student’s t-test by GraphPad Prism (Version 8.4.0). A *P* value less than 0.05 was considered to be significant.

## Results

### QB1561 was highly potent in suppressing drug-resistant cancer cells

We first determined the cytotoxicity of QB1561 by MTS assay in eight cancer cell lines, including the parent human epidermoid carcinoma cell line KB-3-1 and its colchicine-selected ABCB1-overexpressing cell line KB-C2, cisplatin-resistant KCP4 cells (Oiso et al., 2014), ATO-resistant ABCB6 overexpressing KB-ATO cells (Zhang et al., 2017a), human lung cancer cell line NCI-H460 and its mitoxantrone-selected ABCG2-overexpressing NCI-H460/MX20 cells, parent human colorectal cancer cell line SW620 and its adriamycin-selected ABCB1-overexpressing SW620/Ad300 cells.

**FIGURE 2:**
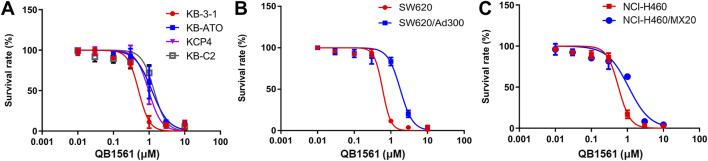
QB1561 was effective in suppressing the proliferation of eight tested cancer cell lines, including KB-3-1, KB-C2, KCP4, KB-ATO **(A)**, SW620 and adriamycin-resistant SW620/Ad300 **(B)**, NCI-H460 and NCI-H460/MX20 **(C)**.

Cell viability curves as shown in Figure 2 and the corresponding IC_50_ values in Table 1 (calculated IC_50_ values of QB1561 and the associated chemotherapeutics that were used as inducers in their corresponding drug-resistant cancer cells) indicated that QB1561 was highly potent in inhibiting the proliferation of all eight cell lines, possessing lower IC_50_ values (0.57–1.80 μM) as compared to AF (0.83–4.23 μM) and certain chemotherapeutics, such as cisplatin, ATO, adriamycin, mitoxantrone, and topotecan in drug-resistant cancer cells. More importantly, QB1561 had much lower resistant profiles since it possessed lower resistance folds (RF), ranging from 1.89 to 3.05, as compared to either AF (RF at around 5) or the original chemotherapeutics used in inducing the resistant cells (Figure 2; Table 1). Typically, the RF of the corresponding chemotherapeutic in drug-resistant cancer cells ranged from 8.45–695, as shown in Table 1 and Supplementary Figure S2, suggesting that they were highly resistant to those chemotherapeutics. In addition, the different inhibitory effects of QB1561 in parent and drug-resistant cancer cells, as shown in Figure 2, which were much less significant than AF and these tested chemotherapeutics, suggested that it might be a weak substrate of ABCB1 and ABCG2. These results indicated that QB1561 can serve as a novel agent to inhibit cancer cells overexpressing ABCB1 and ABCG2.

**TABLE 1:** Calculated IC_50_ values of QB1561 and corresponding chemotherapeutics in different cancer cells.

Cell line	QB1561	AF	Chemotherapeutics
	IC_50_ ^a^ (RF)^b^		IC_50_ (RF)
KB-3–1	0.51 (1)	0.83 (1)	Cisplatin: 1.43 (1); ATO: 2.77 (1); Pac^c^: 0.004 (1)
KCP4	0.97 (1.90)	4.23 (5.10)	Cisplatin: 68.31 (47.77)
KB-ATO	1.20 (2.35)	4.12 (4.96)	ATO 23.4: (8.45)
KB-C2	1.38 (2.71)	ND^d^	Pac: 2.78 (695)
SW620	0.59 (1)	ND	Adriamycin: 0.041 (1)
SW620/Ad300	1.80 (3.05)	ND	Adriamycin: 5.80 (141)
NCI-H460	0.57 (1)	ND	Mitoxantrone: 0.016 (1); Topotecan: 0.059 (1)
NCI-H460/MX20	1.08 (1.89)	ND	Mitoxantrone: 1.07 (66.8); Topotecan: 3.37 (57.1)

^a^ μM.

^b^ RF: Resistance folds as compared to the IC_50_ of parent cells.

^c^ Pac: Paclitaxel.

^d^ ND: not determined.

### QB1561 was able to partially reverse MDR mediated by ABCG2 without altering its expression

**FIGURE 3:**
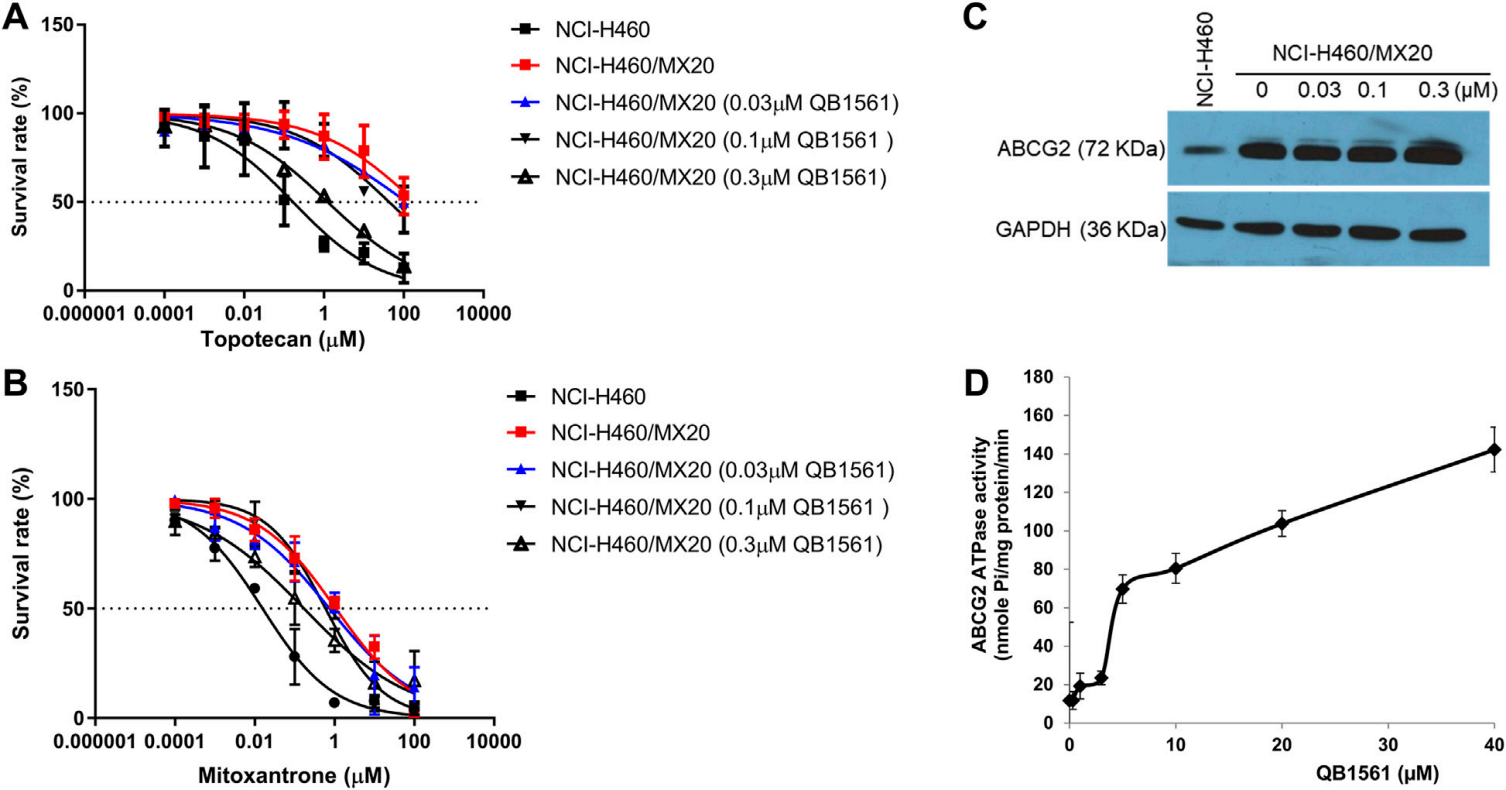
QB1561 could partially reverse ABCG2-mediated MDR in NCI-H460/MX20 cells to topotecan and mitoxantrone without altering the expression of ABCG2. Cell viability of the combination of different concentrations of QB1561 with topotecan **(A)** and mitoxantrone **(B)** in NCI-H460/MX20 cells. **(C)** QB1561 did not affect the expression level of ABCG2. **(D)** QB1561 stimulated the ABCG2 ATPase.

To investigate whether QB1561 could reverse MDR mediated by ABCB1 or ABCG2, we evaluated its effects in combination with paclitaxel, mitoxantrone, and topotecan in KB-C2 and NCI-H460/MX20 cells. QB1561 was tested at three non-toxic concentrations (0.03, 0.1, and 0.3 μM). The results, shown in Figures 3A,B, revealed that QB1561 dose-dependently sensitized NCI-H460/MX20 cells to topotecan and mitoxantrone, both of which are ABCG2 substrates. Specifically, QB1561 at 0.3 μM significantly reduced the IC_50_ of topotecan from 3.37 μM to 0.25 μM and that of mitoxantrone from 1.07 μM to 0.17 μM. However, QB1561 did not fully restore the drug sensitivity to levels observed in parental NCI-H460 cells (Table 1), indicating a partial reversal of ABCG2-mediated MDR. In contrast, QB1561 did not affect the sensitivity of paclitaxel, an ABCB1 substrate, in ABCB1-overexpressing KB-C2 cells (Supplementary Figure S3). Furthermore, the parent compound AF exhibited no reversal effects on MDR mediated by either ABCB1 or ABCG2 (data not shown). Notably, to the best of our knowledge, QB1561 is the first gold(I) complex reported to demonstrate a certain grade of MDR reversal effects. These findings underscore its unique potential as a chemosensitizer for ABCG2 substrates. Future studies are clearly warranted to further explore and optimize QB1561’s ability to sensitize chemotherapeutics against MDR in cancer therapy.

To assess whether QB1561 affected the expression of ABCG2, we treated NCI-H460/MX20 cells with QB1561 at concentrations of 0.03, 0.1, and 0.3 μM for 24 h. The results, shown in Figure 3C, demonstrated that QB1561 did not significantly alter the expression level of ABCG2 under these conditions. However, it remains unclear whether higher concentrations or longer treatment durations could influence ABCG2 expression. Given that ABCG2 is a membrane protein responsible for transporting function, further studies are also needed to investigate whether QB1561 may affect the subcellular localization of ABCG2 on the membrane. This aspect would provide additional insights into whether QB1561’s MDR reversal effect could be mediated by altering the positioning or functionality of ABCG2 at the cell membrane.

We further investigated the effects of QB1561 on ABCG2 ATPase activity, as this enzyme provides ATP for the efflux function of ABCG2 (Wilkens, 2015). As shown in Figure 3D, QB1561 (0–40 μM) significantly stimulated ATPase activity, with a stimulation more than six times greater than the control. This stimulation of ATPase activity could potentially compete with the uptake of ABCG2 substrates, thereby limiting their efflux and enhancing drug retention in the cells. These results suggest that QB1561 sensitizes mitoxantrone and topotecan in NCI-H460/MX20 cells likely by stimulating ATPase activity, rather than through down-regulating ABCG2 expression levels. This mechanism of action provides further evidence for QB1561’s ability to modulate the function of ABCG2 and enhance the efficacy of chemotherapeutics in drug-resistant cancer cells.

### QB1561 reduced the colony formation of ABCG2 overexpressing NCI-H460/MX20 cancer cells

**FIGURE 4:**
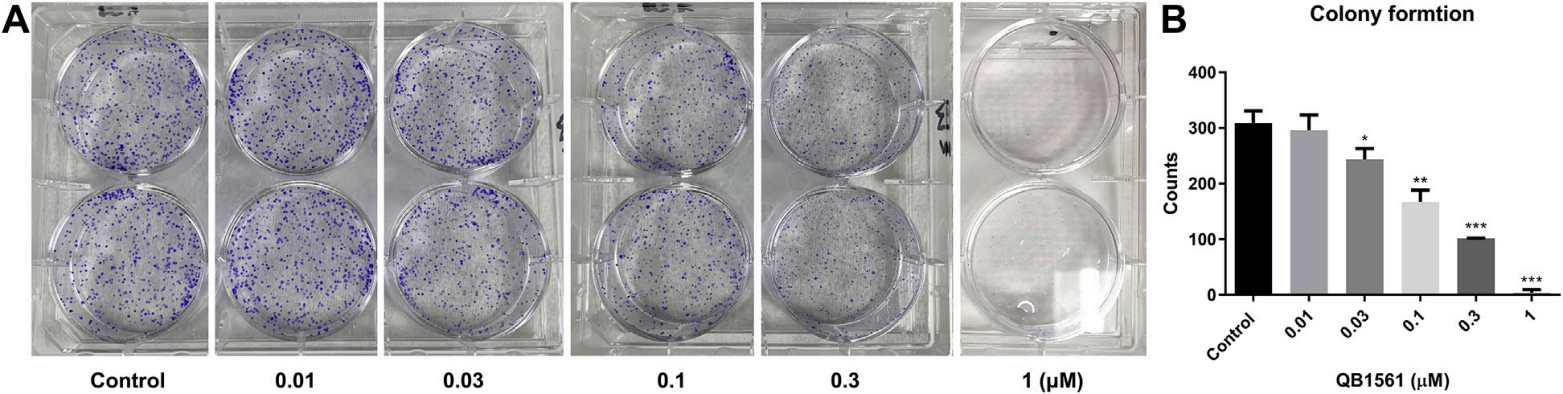
**(A)** Colony formation of NCI-H460/MX20 cells following QB1561 treatment. **(B)** The colony counts of each group. *, **, ***, *P* < 0.05, 0.01, 0.001 *versus* the control group.

We conducted a colony formation assay to further validate the anticancer potential of QB1561. The results demonstrated that QB1561 significantly reduced the colony numbers of NCI-H460/MX20 cells in a concentration-dependent manner, as illustrated in Figures 4A, B. Notably, at a concentration of 1 μM, QB1561 completely inhibited colony formation, effectively eliminating all detectable colonies. This remarkable reduction in colony formation highlights the potent anticancer activity of QB1561 against NCI-H460/MX20 cells. These findings provide additional robust evidence supporting QB1561 as a promising therapeutic agent for targeting this multidrug-resistant cancer cell line.

### QB1561 inhibited cellular TrxR activity and induced the production of ROS

**FIGURE 5:**
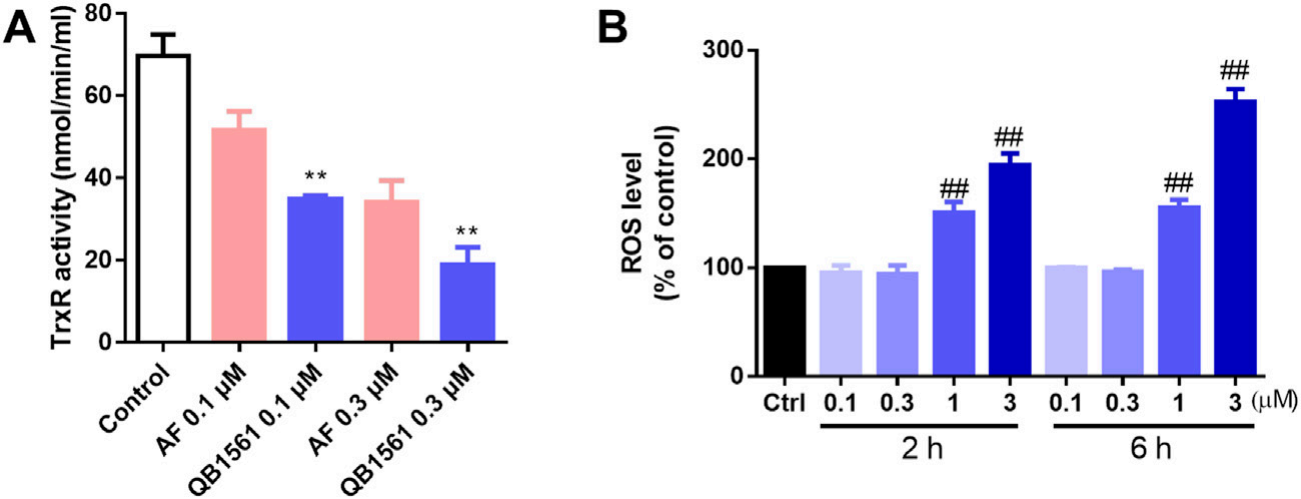
QB1561 inhibited the cellular activity of TrxR **(A)** and induced ROS production in NCI-H460/MX20 cells **(B)**. **P < 0.01 *versus* AF, ^##^ *P* < 0.01 *versus* the control.

The antioxidant TrxR was believed to be the target of gold(I) based complexes including AF and many other dual-gold(I) complexes (Pratesi et al., 2010; Zou et al., 2014; Cui et al., 2024). To further investigate this mechanism, we utilized the TrxR Activity Colorimetric Assay Kit to evaluate the effect of QB1561 on cellular TrxR activity in NCI-H460/MX20 cells. As shown in Figure 5A, treatment with QB1561 at concentrations of 0.1 μM and 0.3 μM for 4 h resulted in a significantly stronger inhibition of TrxR activity compared to the reference compound AF. TrxR, a key protein typically upregulated in drug-resistant cancer cells, plays a critical role in maintaining redox homeostasis by reducing oxidized thioredoxin (Trx) and neutralizing reactive free radicals. The pronounced inhibition of TrxR activity by QB1561 underscores its potential to disrupt redox balance in drug-resistant cancer cells, contributing to its anticancer efficacy. These findings provide valuable insights into the molecular basis of QB1561’s action and its potential as a therapeutic agent for targeting multidrug-resistant cancer (Espinosa and Arner, 2019; Salmain et al., 2023).

To investigate the impact of QB1561 on oxidative stress, the global ROS levels were assessed using the 2′,7′-dichlorodihydrofluorescein diacetate (H2DCFDA) assay. As illustrated in Figure 5B, QB1561 treatment resulted in a marked increase in ROS production at relatively high concentrations (1 μM and 3 μM) over both 2 h and 6 h treatment periods. These findings indicate that QB1561 has the capacity to induce substantial oxidative stress under these conditions. However, further studies are needed to determine whether prolonged treatment at lower concentrations of QB1561 could also lead to ROS induction. The observed inhibition of TrxR activity, coupled with the significant overproduction of ROS, likely contributes synergistically to the cytotoxic effects of QB1561, ultimately promoting cell death of NCI-H460/MX20 cells.

### QB1561 inhibited the mitochondrial respiratory function

**FIGURE 6:**
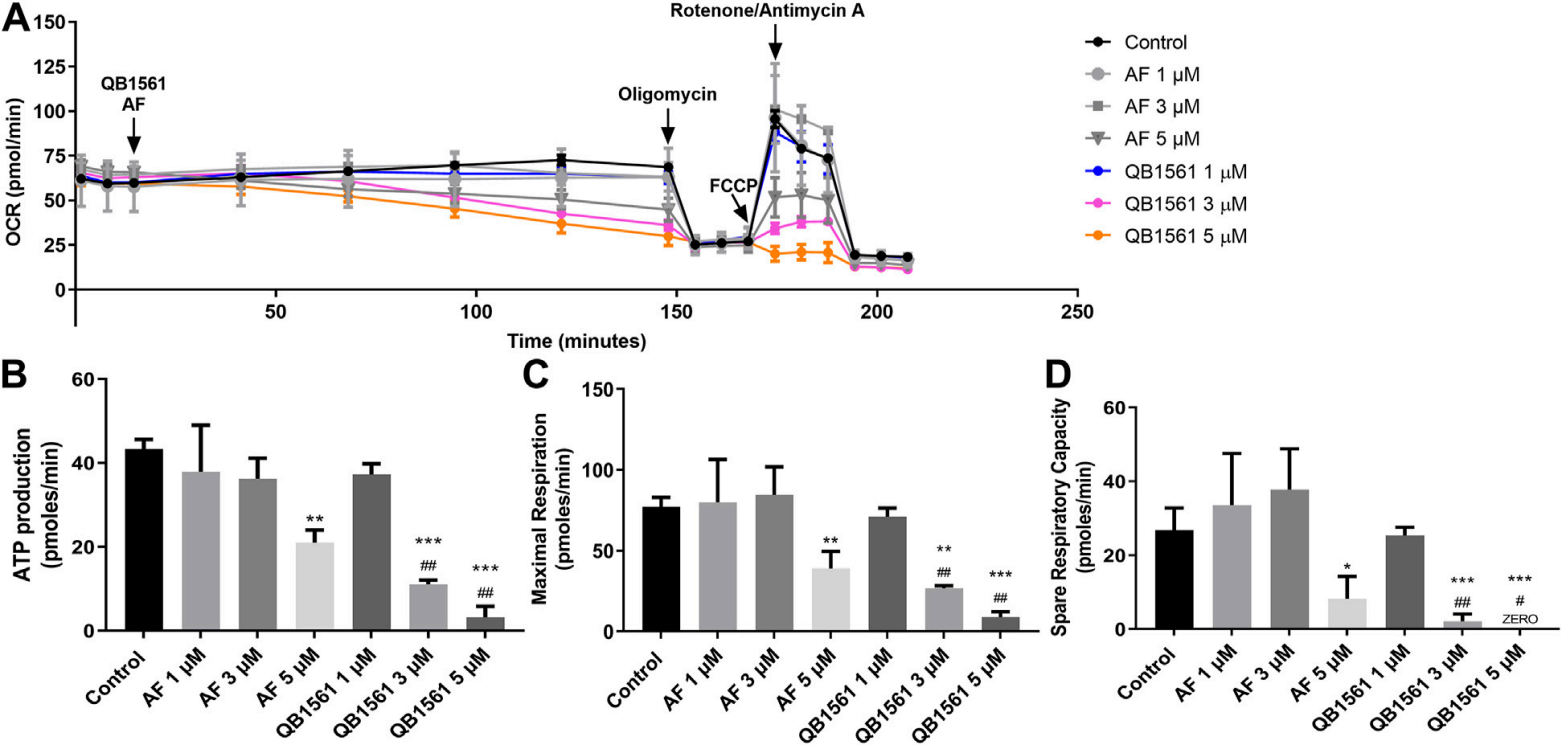
QB1561 targeted and inhibited mitochondrial respiratory functions. **(A)** QB1561 reduced the OCR of mitochondria, leading to remarkably reduced ATP production **(B)**, maximal respiration **(C)**, as well as spare respiration **(D)**. *, **, ***P < 0.05, 0.01, 0.001 *versus* control, respectively. ^#^, ^##^, ^###^ *P* < 0.05, 0.01, 0.001 *versus* the AF group.

Mitochondria in cancer cells, including drug-resistant cancer cells, play essential roles in producing ATP and initiating apoptosis, making them the pivotal players in determining the life or death of cells (Bock and Tait, 2020). The design rationale behind QB1561 is to enhance its ability to target mitochondria and disrupt energy metabolism effectively. To investigate whether QB1561 affects mitochondrial function, we measured the real-time oxygen consumption rate (OCR) in NCI-H460/MX20 cells. OCR serves as a critical indicator of mitochondrial activity, reflecting the efficiency of oxidative phosphorylation and its role in ATP production. Additionally, OCR changes can signify a metabolic shift from oxidative phosphorylation to glycolysis, a universal hallmark of metabolic reprogramming in cancer cells. By analyzing these parameters, we aimed to elucidate the impact of QB1561 on mitochondrial function and its potential to interfere with the energy metabolism of drug-resistant cancer cells (Muller et al., 2019). The results, as shown in Figures 6A–D, revealed that QB1561 significantly reduced the OCR compared to both the control group and AF. Specifically, the data calculated from OCR measurements indicated that QB1561 exhibited a stronger inhibitory effect on mitochondrial functions, including ATP production (Figure 6B), maximal respiration capacity (Figure 6C), and spare respiratory capacity (Figure 6D). These findings suggest that QB1561 effectively targeted mitochondrial function, disrupting its respiratory efficiency and energy production. As the Trx system exists in the cytosol, nucleus and mitochondria, it would be reasonable to assume that QB1561 also affected the Trx system in mitochondria, further resulting in increased production of ROS. In comparison, the gold compound AF is known to effectively inhibit mitochondrial TrxR2 and Trx (Du et al., 2012). Detailed studies on the mechanism of inhibition of the Trx system by QB1561 could perhaps explain at least one enzymatic mechanism by which QB1561 caused mitochondrial dysfunction.

The above results indicate that AB1561 suppressed OCR, which then contributes to the induction of cell death. However, it remains to be determined whether the cells compensate for this mitochondrial dysfunction by activating glycolysis. Further studies are required to explore this potential compensatory metabolic shift and its implications for the overall mechanism of action of QB1561. These results underscore the potential of QB1561 as a mitochondrial-targeting agent for combating drug-resistant cancer cells.

## Discussion

The findings of the current study demonstrate that QB1561, a novel metal complex containing two gold (Au) atoms, functions as a multi-faceted anticancer agent. Firstly, QB1561 exhibited remarkable potency in suppressing the proliferation of cancer cells overexpressing ABC transporters, outperforming both the parent drug AF and certain clinically utilized chemotherapeutics that are substrates of ABCB1 or ABCG2. This highlights QB1561’s superior efficacy against drug-resistant cancer cells overexpressing ABC transporters. Importantly, QB1561 showed lower IC_50_ values in parent cancer cell lines with minimal ABC transporter expression compared to resistant cells, suggesting that it may be a weak substrate of ABCB1 or ABCG2. However, it is worth noting that prolonged treatment with QB1561 may potentially upregulate the expression of ABCB1 and ABCG2, thereby inducing resistance to QB1561 over time. This highlights the need for further investigations into strategies to prevent or counteract the development of resistance during long-term treatment. These findings establish QB1561 as a promising therapeutic candidate with notable advantages over existing chemotherapeutics for treating drug-resistant cancers mediated primarily by ABC transporters.

Second, QB1561 at low concentrations, could partially reverse resistance of mitoxantrone or topotecan mediated in ABCG2-overexpressing NCI-H460/MX20 lung cancer cells, suggesting it can serve as a new ABCG2 regulator. The inhibition of ABCG2 can be attributed to the downregulated expression level in cells, which QB1561 did not alter. Interestingly, QB1561 could stimulate the ABCG2 ATPase, which can bind the nucleotide-binding domain (NBD) of ABCG2, suggesting that QB1561 may competitively limit the uptake of the substrate of ABCG2, leading to decreased efflux of mitoxantrone or topotecan, which needs further studies. In addition, further studies/applications are warranted by exploring other combinational regimens and in different cell lines that overexpress ABCG2.

Third, QB1561 could dramatically induce the production of ROS via dual inhibition of TrxR and mitochondrial respiration. In cancer cells, well-balanced redox homeostasis is quite critical for cell survival, and the disturbance of redox homeostasis can lead to cell death (Cui et al., 2018a). Redox homeostasis, maintained by the production and elimination of ROS, regulates multiple cellular processes such as cell proliferation and pro-survival signaling cascades, and the key players in regulating redox homeostasis can serve as targets for anticancer agents (Cui et al., 2018b; Trachootham et al., 2009). TrxR is a key enzyme in cancer cell biology, which is also one of ROS eliminating proteins (Zhang et al., 2017b). TrxR is upregulated in drug-resistant cancer cells and certain types of cancer patients (Cho et al., 2019), suggesting it is a druggable target as confirmed by various TrxR inhibitors including AF under clinical trials (Duan et al., 2016; Sze et al., 2019). Mitochondria in cancer cells are the major source of ROS, and its further disturbance by Mitocans, a term of agents that can target mitochondria (Dong et al., 2020), can cause overproduction of ROS, which may finally lead to overloaded oxidative stress and cell death. In this study, we found that QB1561 could (1) concentration-dependently inhibit the cellular activity of TrxR, and (2) target and impact mitochondria, both of which could induce significant production of ROS, which may eventually lead to cell death. QB1561 could paralyze the respiratory function of mitochondria as it markedly suppressed the OCR, leading to decreased oxidative phosphorylation and ATP production. However, it is still inconclusive whether glycolysis is activated by cancer cells after QB1561 treatment, and more studies are required. Additionally, since the Trx system is also present in mitochondria, it is worth exploring whether QB1561 can inhibit mitochondrial TrxR.

While the current study provides significant insights into QB1561’s anticancer potential, several limitations warrant further investigation. First, the potential application of QB1561 as a reversal agent of MDR mediated by ABCG2 should be explored through studies involving various drug combinations. Additionally, the impact of QB1561 on the efflux function of ABCG2 requires clarification to understand fully its interaction with this transporter. Although QB1561 has been suggested to target mitochondria, further *in vitro* experiments are needed to study its cellular distribution and subcellular localization. Furthermore, the specific binding targets of QB1561 remain unidentified, necessitating structural and biochemical studies to uncover these interactions. The underlying mechanisms of cell death induced by QB1561 also remain to be elucidated, particularly with respect to signaling pathways such as apoptosis and the adaptation of energy metabolism. Finally, as a leading compound, the *in vivo* potency of QB1561 should be validated. This includes evaluating its efficacy in xenograft models using both sensitive and drug-resistant cancer cell lines. Addressing these gaps will be critical to advancing QB1561 from a promising candidate to a viable therapeutic agent.

In summary, the findings of this study not only support the further evaluation of QB1561 as a novel agent to combat drug-resistant cancers, but also offer valuable insights for the broader field of gold-based anticancer therapeutics. Specifically, the information gathered provides (1) a deeper understanding of the action mode of gold complexes. This study highlights the unique properties and mechanisms of complexes containing two gold (Au) atoms, contributing to the growing knowledge of gold-based compounds in cancer treatment. (2) Inspiration for advancing gold complex development. By demonstrating the multifunctional anticancer activity of QB1561, this research underscores the potential of gold complexes as promising candidates for drug discovery, encouraging further exploration in this area. And (3) guidance for designing novel gold complexes. This study suggests opportunities to refine the design of gold-based compounds by modifying key components, including thiol donors, Au atom number, and phosphine ligands, to enhance their efficacy and selectivity. Overall, the findings set the stage for continued innovation in gold complex-based anticancer drug discovery, with QB1561 serving as a valuable lead compound for future research.

## Conclusion

The dual-gold complex QB1561 partially reversed MDR mediated by ABCG2 and effectively suppressed the proliferation of drug-resistant cancer cells overexpressing ABCB1 and ABCG2. This anticancer activity was achieved through a dual inhibition of TrxR and mitochondrial respiration. These dual actions highlight QB1561 as a promising therapeutic agent for drug-resistant cancer cells.

## Data Availability

The original contributions presented in the study are included in the article/Supplementary Material, further inquiries can be directed to the corresponding authors.
