# Supplementary material for: Gold complex QB1561 suppresses drug-resistant cancer cells by inhibiting TrxR and mitochondrial respiratory function
Source: Front Pharmacol. 2025 Feb 24;16:1560880. doi: 10.3389/fphar.2025.1560880 (PMC11891169; doi:10.3389/fphar.2025.1560880)
Supplement: Supplementary file 1 [file DataSheet1.pdf]

Supporting Materials

Gold complex QB1561 suppresses drug-resistant cancer cells by inhibiting TrxR and mitochondrial respiratory function

Hai-Ling Gao,[1] Wenwen Ding,[2] Zhi-Xin Shen,[3*] Qingbin Cui,[2,4*]

[1]Department of Histology and Embryology, Shandong Second Medical University, Weifang, Shandong 261053, China. [2]Department of Experimental Research, Sun Yat-sen University Cancer Center, Guangzhou, China. [3]Department of Thyroid and Breast Surgery, Affiliated Hospital of Shandong Second Medical University, Weifang, Shandong 261042, China. [4]Current affiliation and address: Department of Cell and Cancer Biology, University of Toledo College of Medicine and Life Sciences, Toledo, OH 43614, US.

* Correspondence: Zhi-Xin Shen, szx1758@163.com; Qingbin Cui, Qingbin.cui@utoledo.edu

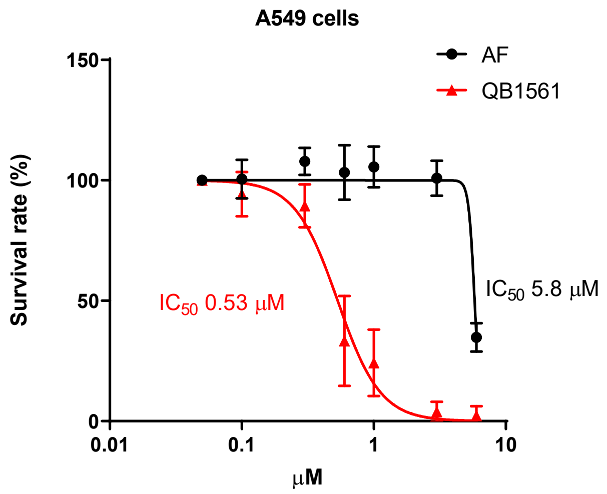

FIGURE S1. The cell survival curve of QB1561 and AF in A549 lung cancer cells.

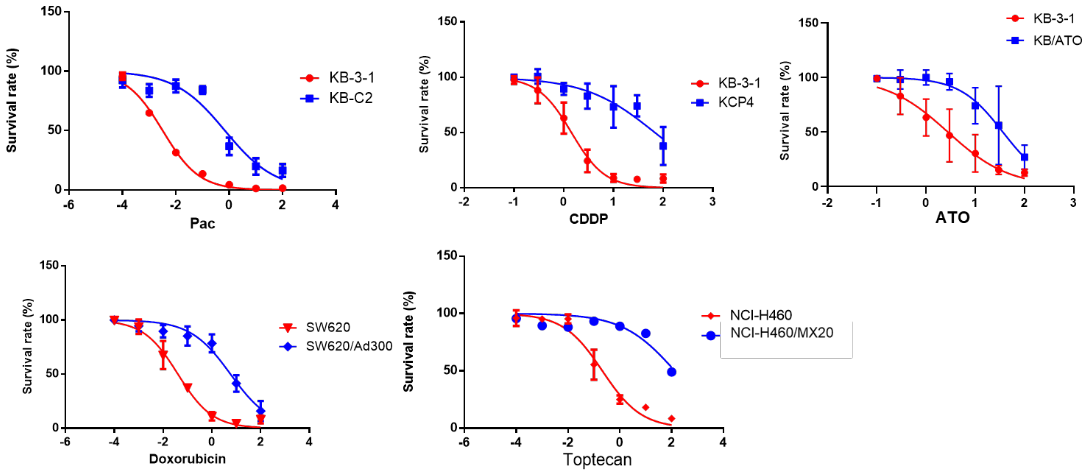

FIGURE S2. Adriamycin, paclitaxel, ATO, topotecan, CDDP showed resistant profiles in their corresponding resistant cancer cell lines.

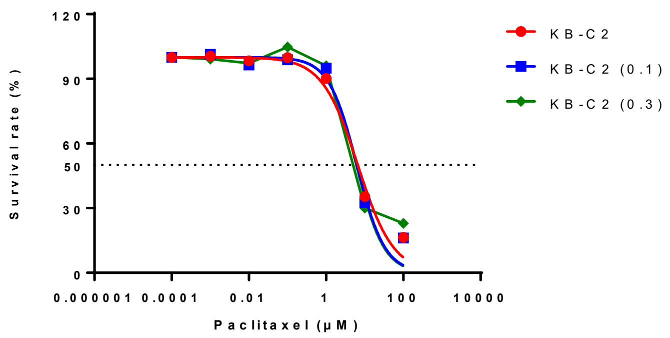

FIGURE S3. QB1561 (0.1 and 0.3 μM) didn't alter the sensitivity of paclitaxel which is an ABCB1 substrate.

H-NMR of QB1561

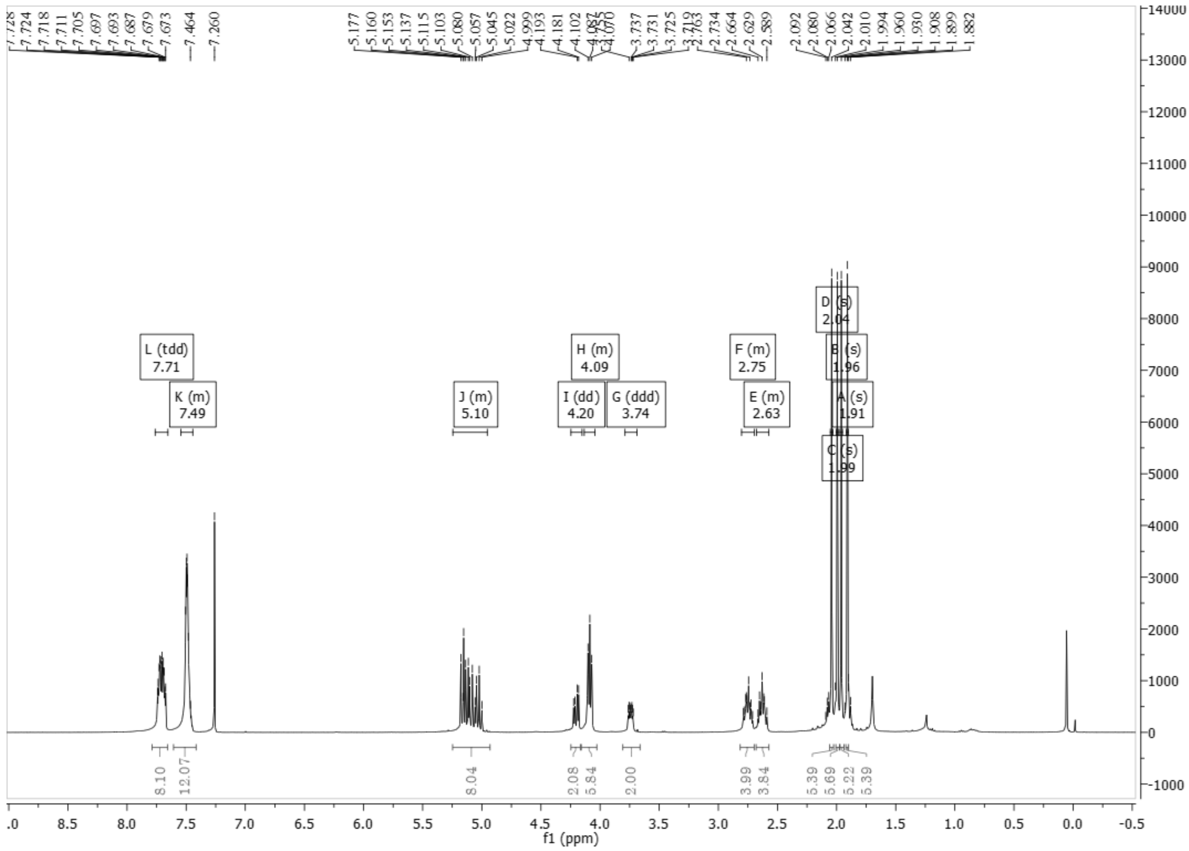

C-NMR of QB1561

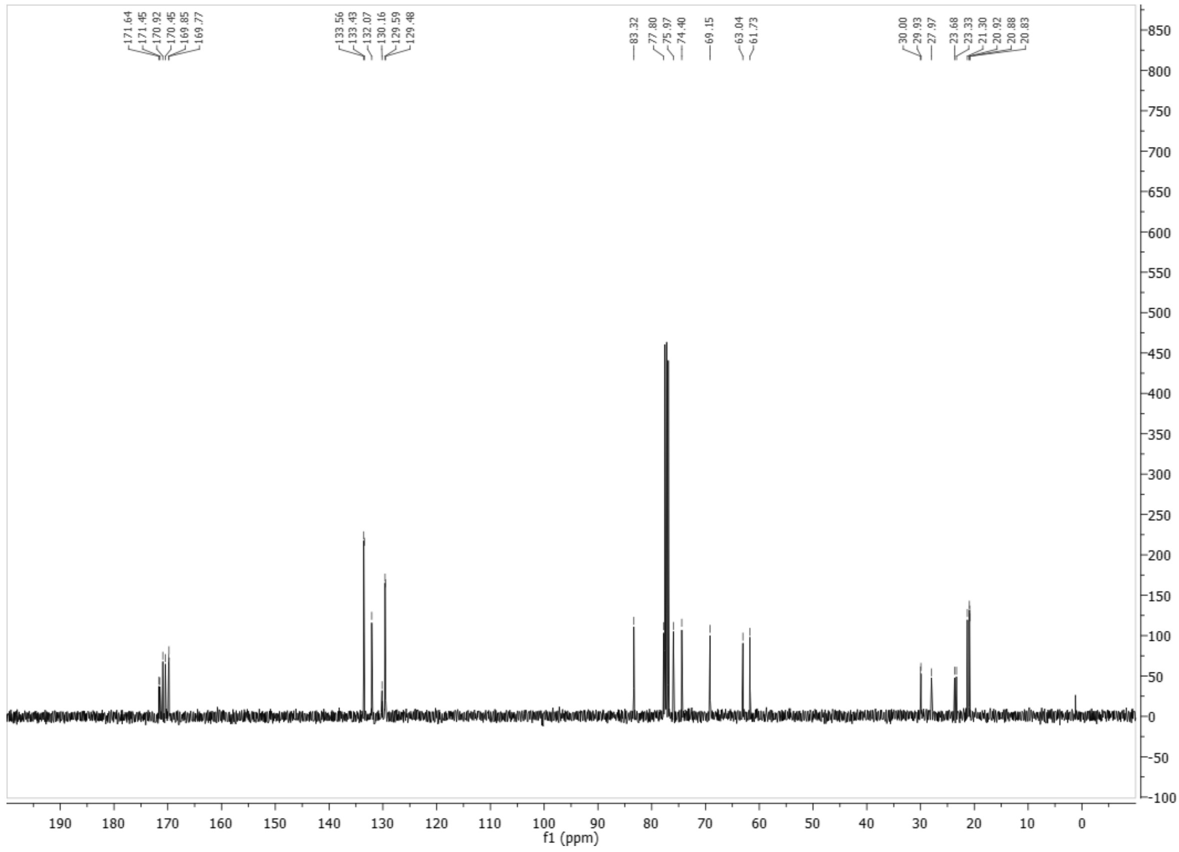

P-NMR of QB1561

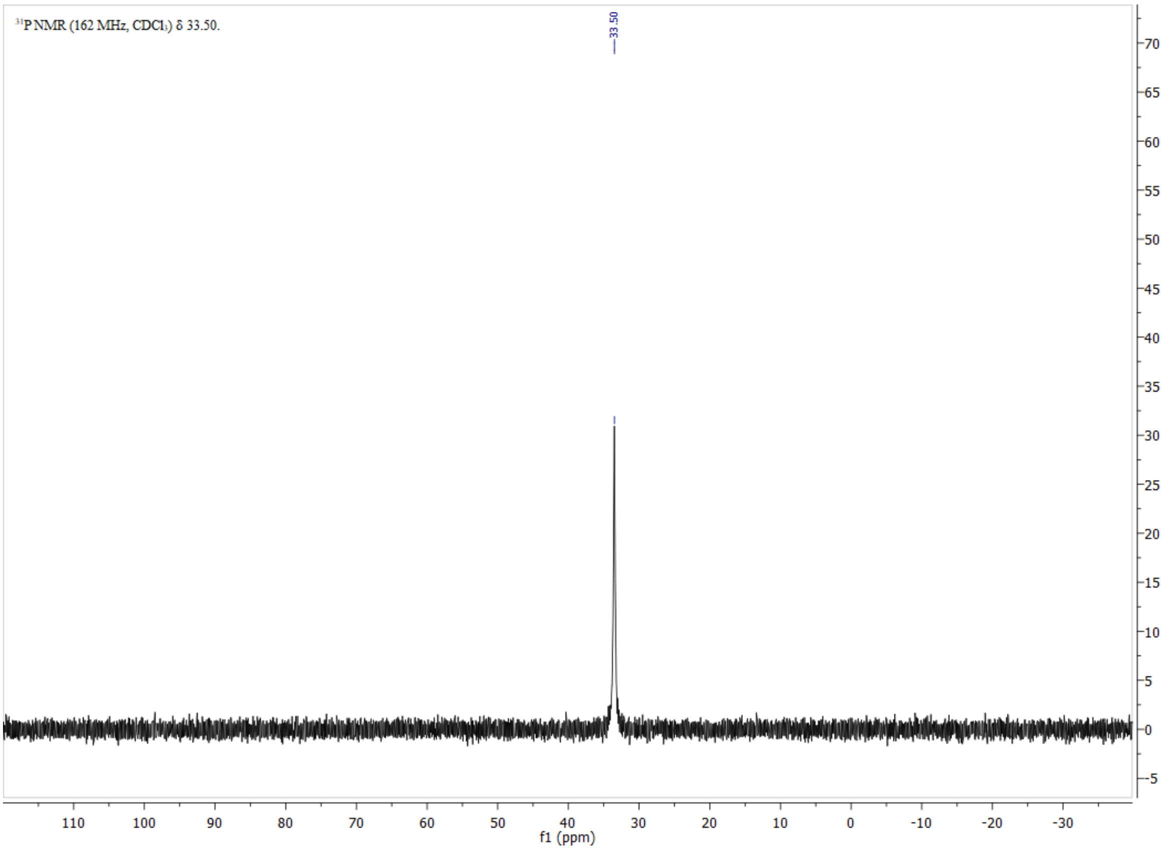

QB1561: ^{1}H NMR (400 MHz, $CDCl_3$) δ 7.71 (tdd, J = 9.5, 5.4, 2.1 Hz, 8H), 7.57–7.41 (m, 12H), 5.23–4.91 (m, 8H), 4.20 (dd, J = 12.2, 4.9 Hz, 2H), 4.13–4.01 (m, 6H), 3.74 (ddd, J = 9.5, 4.8, 2.4 Hz, 2H), 2.82–2.70 (m, 4H), 2.69–2.57 (m, 4H), 2.04 (s, 6H), 2.00 (s, 6H), 1.96 (s, 6H), 1.90 (s, 6H). ^{13}C NMR (101 MHz, $CDCl_3$) δ 171.64, 171.45, 170.92, 170.45, 169.85, 169.77, 133.56, 133.43, 132.07, 130.16, 129.59, 129.48, 83.32, 77.80, 75.97, 74.40, 69.15, 63.04, 61.73, 30.00, 29.93, 27.97, 23.68, 23.33, 21.30, 20.92, 20.88, 20.83. ^{31}P NMR (162 MHz, $CDCl_3$) δ 33.50.
